# Impact of an enhanced recovery pathway on length of stay and complications in elective radical cystectomy: a before and after cohort study

**DOI:** 10.1186/s13741-019-0120-4

**Published:** 2019-08-22

**Authors:** W. Jonathan Dunkman, Michael W. Manning, John Whittle, John Hunting, Edward N. Rampersaud, Brant A. Inman, Julie K. Thacker, Timothy E. Miller

**Affiliations:** 1 Division of General, Vascular and Transplant Anesthesia, Duke University, Durham, NC USA; 2 Department of Anesthesiology, Duke University, Durham, NC USA; 3 Division of Urology, Duke University, Durham, NC USA; 4 Division of Surgical Oncology, Duke University, Durham, NC USA

**Keywords:** Cystectomy, Enhanced recovery, ERAS, Fluid management

## Abstract

**Background:**

Enhanced recovery after surgery (ERAS) pathways aim to standardize and integrate perioperative care, incorporating the best available evidence-based practice throughout the perioperative period targeted at attenuating the surgical stress response while optimizing physiologic function, with the goal of facilitating recovery. Radical cystectomy is associated with significant postoperative morbidity, but comprehensive ERAS pathways have not been well studied in this population.

**Methods:**

This is a before and after cohort study of an ERAS pathway for radical cystectomy at a large academic medical center. Following introduction of the ERAS pathway and a wash in period, we prospectively collected data from the next 100 consecutive subjects undergoing radical cystectomy with the ERAS pathway. This cohort was compared to a retrospective cohort of 100 consecutive patients undergoing radical cystectomy with traditional care. The primary outcome was hospital length of stay. Secondary outcomes included perioperative management, time to recovery milestones, complications, and costs.

**Results:**

Implementation of an ERAS pathway for radical cystectomy was associated with reduced hospital length of stay (median LOS 10 days (IQR = 8–18) vs 7 days (IQR = 6–11); *p* < 0.0001), reduced time to key recovery milestones, including days to first stool (5.83 vs 3.99; *p* < 0.001) and days to first solid food (9.68 vs 3.2; *p* < 0.001), reductions in some complications, and a 26.6% reduction in overall costs (*p* < 0.001).

**Conclusions:**

Our data support the use of an ERAS pathway for radical cystectomy and add to the increasing body of literature supporting enhanced recovery over a wide variety of procedures.

**Trial registration:**

Not applicable.

## Background

Enhanced recovery after surgery (ERAS) refers to surgery-specific pathways that are patient-centered and multidisciplinary and which aim to standardize and integrate a range of perioperative interventions throughout the perioperative period while incorporating the best available evidence-based medicine. ERAS pathways seek to attenuate the surgical stress response and to optimize physiologic and organ function, and in doing so achieve early recovery (Kehlet and Wilmore 2008; Ljungqvist et al. 2017).

Things which can prevent hospital discharge after surgery are the need for parenteral analgesia, gut dysfunction with consequent and perhaps contributory intravenous fluid administration, and lack of mobility (Kehlet and Wilmore 2008; Ljungqvist et al. 2017; Miller et al. 2014). These factors often overlap and interact to delay recovery and discharge from the hospital. The fundamental elements of an ERAS pathway are designed to target these issues and address the pre-, intra-, and post-operative phases of the patients’ journey.

Many of the early efforts at organized ERAS pathways targeted colorectal surgery and have been shown to be highly successful at reducing the length of stay and complications after such operations (Miller et al. 2014; Wind et al. 2006; Khoo et al. 2007; Lassen et al. 2009). Many of the same issues and challenges faced by patients undergoing colorectal surgery are also experienced by patients undergoing other major abdominal surgeries, including radical cystectomy. ERAS guidelines and recommendations for radical cystectomy were published in 2013. However, the guidelines noted a paucity of studies addressing ERAS for cystectomy patients and called for more data and a comprehensive protocol to guide future care (Cerantola et al. 2013).

Despite the increasing evidence in support of elements of ERAS care pathways and success with early versions of these pathways, good quality data from comparative studies of comprehensive, modern ERAS protocols with information on length of stay, recovery, complications, and costs is still lacking. Many of the existing series are based on older or less comprehensive enhanced recovery pathways which may not show the full benefit of more complete, modern pathways. In addition, some of the series report good results but do not offer a control group for comparison. Many do not provide much detail regarding complications and have not analyzed the costs associated with implementing these programs.

We sought to carefully analyze the effect of implementing a full enhanced recovery program for radical cystectomy on length of stay, recovery, complications, and costs.

## Methods

This study was approved by the Duke University Medical Center institutional review board (IRB# Pro00052995) and is a before and after cohort study of a single-center, quality improvement project to evaluate the effectiveness of a full ERAS pathway in radical cystectomy when compared to a historical cohort of patients undergoing radical cystectomy with traditional care. After introduction of the ERAS pathway for radical cystectomy, we allowed a wash in period for training and familiarization with the pathway. Originally intended to be 3 months, this wash in period became approximately 1 year, or 50 patients, as we prepared to enroll patients. Following this period, we collected data from the next 100 consecutive subjects undergoing radical cystectomy with the ERAS pathway (January 14, 2015, to October 23, 2017). Data was considered under an intention-to-treat analysis. Data on these subjects was collected prospectively and by review of the electronic medical record. Informed consent was obtained from these patients during the preoperative evaluation process.

The prospective ERAS cohort was compared to a historical cohort of 100 consecutive patients undergoing radical cystectomy with traditional care prior to the implementation of the ERAS pathway (January 14, 2011, to August 16, 2013). All data on these patients was collected retrospectively, and the IRB waived the requirement for informed consent for this group.

Data collected for both groups included intraoperative management, pain scores, opioid use, complications, time to recovery milestones, time to discharge, readmissions, and costs (Table 1). Cost data was obtained from the hospital business office.

**Table 1:** Data collected

Intraoperative fluids, opioid requirements, estimated blood loss, and urine output	
Average pain score in PACU and twice a day after that until discharge	
Opioid consumption in PACU and every day until discharge	
Intravenous fluid given in PACU and each day postoperatively	
Use of interventions for hypotension such as fluid boluses or vasopressors	
Complications: pneumonia, respiratory failure, wound infection, urinary tract infection, heart failure, myocardial infarction, deep vein thrombosis, pulmonary embolus, cardiac arrhythmia, renal dysfunction, postoperative ileus, anastomotic leak, sepsis, confusion/delirium, nausea/vomiting	
Daily physical activity and sleep duration and quality	
Date and time of first bowel movement	
Time until tolerating solid foods	
Time to hospital discharge	
Readmission, including causes	
Hospital costs	

The primary outcome of this study is hospital length of stay (LOS) after surgery, defined as the number of postoperative nights in the hospital. Secondary outcomes are time to first bowel movement, time to ingestion of solid food, estimated blood loss, postoperative pain scores, postoperative opioid requirements, complications, readmissions, and hospital costs. The definition of ileus can be quite variable, and for our study was defined based on the clinical judgment of the primary team at the time of care.

### Traditional care

Patients in the traditional care group were cared for according to provider preference as there was no standardized pathway between surgeons or anesthesiologists before initiation of ERAS. Our institution had implemented an enhanced recovery pathway for colorectal surgery in the years prior to the historical period for this study. The same pool of anesthesiologists covers these cases, and it is possible that some of the principles of enhanced recovery may have carried over into their intraoperative management. For example, epidural analgesia was common but not universal or standardized. Multimodal analgesics were given at provider discretion and were not common. General principles of restrictive fluid management were common by this time, but advanced hemodynamic monitors were used in a minority of cases and goal-directed fluid therapy was not practiced in an organized way. The pre- and post-operative elements of the enhanced recovery pathway were not in place. Many of the potential advantages related to the intraoperative management may not have been fully realized if patients were not prepared preoperatively and encouraged to ambulate and eat early postoperatively.

### The ERAS pathway

Patients were cared for according to the ERAS pathway described in Table 2. This pathway is now the standard of care for all patients undergoing radical cystectomy at Duke University Medical Center after January 13, 2014. The pathway features preoperative education, multimodal analgesia, thoracic epidural, optimal fluid management, and early mobilization and PO intake after surgery. For patients in this study, we used the EV1000 cardiac output monitor (Edwards Lifesciences, Irvine, CA) with the FloTrac sensor connected to the arterial line for cardiac output monitoring in the goal-directed fluid therapy component of this pathway (Table 2).

**Table 2:** Enhanced recovery for radical cystectomy pathway

Preoperative management	
Patient educated about the pathway in the surgical clinic	
Preoperative bowel preparation is not routinely used	
Patients allowed clear fluids until 2 h before the start of surgery	
500 mL carbohydrate drink 2 h before surgery (Clearfast)	
Oral adjunctive analgesics given preoperatively: acetaminophen, gabapentin	
Alvimopan given preoperatively	
Transdermal scopolamine patch applied in preoperative holding unless contraindicated	
Low thoracic epidural placed with small amount of IV fentanyl and/or midazolam for sedation	
Heparin 5000u SC given after epidural placement and before incision	
Antibiotic prophylaxis: cefazolin or clindamycin if penicillin allergic	
Intraoperative management	
Induction: lidocaine, propofol, fentanyl up to 150 μg, neuromuscular blocking drug of choice	
Goal is to avoid IV opioids, no IV opioids after induction without discussion with attending anesthesiologist	
Dexamethasone 4 mg IV after induction	
ASA standard monitors and arterial line with cardiac output monitor	
Volatile anesthetic titrated to keep BIS 40–60	
Option for epidural hydromorphone 0.4 mg at induction	
Epidural infusion bupivacaine 0.0625–0.25% ± hydromorphone 10 μg/mL run at 3–6 mL/h	
Ketamine infusion 4 μg/kg/min may be used in chronic pain patients	
Ondansetron 4 mg IV given at the end of surgery	
Acetaminophen 1 g IV and ketorolac 15 mg IV given towards end of the case if appropriate	
Fluid management:	
Maintenance crystalloid infusion (LR) 3 mL/kg based on ideal body weight	
Goal-directed fluid therapy—colloid boluses to maximize stroke volume	
Record initial stroke volume (SV)	
After incision, give 250 mL colloid bolus over < 15 min	
If SV increases by > 10%, repeat bolus	
If SV increases by < 10%, patient does not require a further bolus	
Record peak value achieved	
If still hypotensive, consider phenylephrine bolus or infusion	
Give a further colloid bolus when SV drops 10% from peak value	
Repeat cycle	
Blood products transfused as needed	
Postoperative management	
Epidural bupivacaine 0.0625–0.125% ± hydromorphone 10 μg/mL run at 4–6 mL/h for up to 72 h	
(Hydromorphone 10 μg/mL alone may be used in hypotension is a problem)	
Scheduled adjunctive analgesia with acetaminophen and NSAIDs whenever possible	
Patients transitioned to oral opioids after removal of epidural catheter	
Patients encouraged to drink liquids immediately after surgery	
Alvimopan given postoperatively for 5 days or until first stool	
IV fluids discontinued once adequate oral intake is achieved, usually the first morning after surgery	
All preoperative medications are restarted when patients tolerate oral intake	
Patients cared for in an environment that encourages early mobilization	
Encouraged to be out of bed on the day after surgery and for at least 6 h on every subsequent day	
Patients are asked to maintain a diary of their activity and sleep	

### Statistical methods

Using recent data available at the time of the study preparation, the average LOS after cystectomy was 15 ± 13 days. It was determined that a sample size of 98 patients per group would achieve 80% power to detect a difference in LOS of 4 days at alpha = 0.05. As such, it was decided to study a total of 100 patients per group.

Descriptive statistics were calculated for demographics and comorbidities for both cohorts and differences tested for statistical significance. Continuous variables were presented as either mean and standard deviation or median and interquartile range and tested with either the two-sample *t* test or Wilcoxon rank-sum test depending on their distribution. Categorical variables were presented as count and frequency and tested using either chi-squared test or Fisher’s exact test as appropriate.

As the two cohorts were significantly different regarding smoking history, an adjusted analysis was performed with smoking status dichotomized into never smoked vs ever smoked. Length of stay was determined to be non-normal and was non-normal after log transformation. To compare the length of stay between groups, a linear regression model was built with the independent variable length of stay and the dependent variable smoking history. The residuals from the model were then compared between the two cohorts using the Wilcoxon rank-sum test. This method was repeated for the additional continuous outcomes.

Dichotomous outcomes, including adverse events, were compared between the two cohorts using multivariable logistic regression models. Each outcome of interest was fit with the independent variables ERAS protocol and smoking history.

Several of the day-to-event outcomes had varying levels of missing data due to the nature of the variable and the reported means are among those with data and the adjusted measure of association is among those with data.

Pain was assessed using a 0–10 verbal response scale (VRS), where 0 represents “no pain” and 10 represents “the worst possible pain,” twice a day as part of the standard of care nursing protocol. The highest pain reported each day was recorded from the day of surgery until discharge or the fifth postoperative day. Opioid use was converted to and compared as morphine equivalents.

The EV1000 cardiac output monitor allows for the definition of target stroke volume (SV) and tracking of percentage time within that target. The time in target variables were recorded among ERAS patients. A supplementary analysis was performed to determine the relationship between SV time in target and outcomes of interest. For the continuous outcome variables of interest, Spearman’s correlation coefficients were calculated between the time in target variable and the outcome of interest. For the dichotomous outcomes, univariable logistic regression models were fit between the time in target variables and the outcome of interest.

Statistical significance was specified as *p* < 0.05. SAS 9.4 (SAS Inc., Cary, NC) was used for all analysis. This manuscript adheres to the STROBE guidelines for cohort studies and the RECOvER checklist for Reporting on ERAS Compliance and Outcomes Research (Elias et al. 2018).

## Results

There were 100 patients in the historical control cohort and 100 patients in the ERAS cohort. Patient characteristics for each group are summarized in Table 3. There were no significant differences in age, gender, BMI, ASA, or comorbidities. There were significantly fewer smokers in the ERAS group, perhaps reflecting a population level decrease in smoking between the two time periods, which was controlled for in the rest of the analyses (Table 3).

**Table 3:** Patient characteristics

	Historical control (*N* = 100)	ERAS patients (*N* = 100)	*p* value
Gender (male)	79 (79.00%)	69 (69.00%)	0.107^1^
Age	68.00 [69.75, 77.00]	66 [61.00, 72.25]	0.360^2^
BMI (kg/m^2^)	26.88 [24.22, 30.47]	27.83 [24.83, 31.58]	0.567^2^
ASA class			0.331^1^
ASA 1	0 (0.00%)	1 (1.00%)	
ASA 2	12 (12.00%)	7 (7.00%)	
ASA 3	81 (81.00%)	88 (88.00%)	
ASA 4	7 (7.00%)	4 (4.00%)	
Smoker			0.017^1^
Current smoker	20 (20.00%)	8 (8.00%)	
Former smoker	62 (62.00%)	62 (62.00%)	
Never smoker	18 (18.00%)	30 (30.00%)	
Diabetes mellitus	27 (27.00%)	25 (25.00%)	0.747^1^
Diabetes control			0.241^1^
Medically treated	25 (96.15%)	20 (86.96%)	
Diet controlled	1 (3.85%)	3 (13.04%)	
History of cardiac disease	68 (68.00%)	72 (72.00%)	0.537^1^
History of respiratory disease	42 (42.00%)	39 (39.00%)	0.666^1^
Preoperative hemoglobin (g/dL)	11.20 [9.70, 13.05]	12.00 [10.10, 13.30]	0.155^2^
Preoperative white blood cell count (g/dL)	8.30 [6.60, 11.20]	7.70 [6.15, 9.85]	0.112^2^
Preoperative creatinine (g/dL)	1.20 [0.95, 1.70]	1.10 [0.90, 1.40]	0.075^2^

Continuous variables reported as median [Q1, Q3]; categorical variables reported as count and percent

History of cardiac disease includes MI, angina, pulmonary edema, cardiac treatment, peripheral edema, and other unspecified

History of respiratory disease includes chronic obstructive pulmonary disease, asthma, mild or limiting dyspnea, dyspnea at rest, home oxygen use, sleep apnea, and other unspecified

*p* value key: ^1^Chi-square, ^2^Wilcoxon

Overall compliance with the ERAS protocol was quite high (95–100% for many elements) (Table 4). Use of a preoperative carbohydrate drink was entirely novel in the ERAS group and had good compliance (87%). PONV and thrombosis prophylaxis were common in the historical control group but increased in the ERAS group (to 98% and 97% respectively). Epidural use increased from 74 to 95%. Use of an advanced hemodynamic monitor increased from 13 to 99%. Postoperative use of a nasogastric tube decreased from 55 to 30%.

**Table 4:** Intraoperative management

	Historical controls (*N* = 100)	ERAS patient (*N* = 100)	*p* value
ERAS pathway compliance			
Preoperative oral carbohydrate drink	0 (0.00%)	87 (87.00%)	< 0.001^1^
Unknown	0 (0.00%)	10 (10.00%)	
Antibiotic prophylaxis in the OR	98 (98.00%)	100 (100.00%)	0.497^1^
Thrombosis prophylaxis	62 (62.00%)	97 (97.00%)	< 0.001^1^
Epidural placed	74 (74.00%)	95 (95.00%)	< 0.001^1^
Any intraoperative block	4 (4.00%)	2 (2.00%)	0.683^1^
PONV prophylaxis administered	80 (80.00%)	98 (98.00%)	< 0.001^1^
Number of PONV prophylaxis medications given	0.81 (0.46)	1.37 (0.54)	< 0.001^2^
Upper-body forced-air heating cover used	99 (99.00%)	99 (99.00%)	1.000^1^
Hemodynamic monitor	13 (13.00%)	99 (99.00%)	< 0.001^1^
Unknown	11 (11.00%)	0 (0.00%)	
Nasogastric tube used postoperatively	55 (55.00%)	30 (30.00%)	< 0.001^3^
Parenteral opioids given within 48 h postoperatively	32 (32.00%)	39 (39.00%)	0.301^3^
Fluids			
Intraoperative blood loss (mL)	1411.25 (901.84)	999.25 (549.87)	0.001^2^
RBC transfusion	80 (80.00%)	48 (48.00%)	< 0.001^3^
RBC transfusion amount (mL)	945.47 (917.51)	408.26 (544.82)	< 0.001^2^
FFP transfusion	27 (27.00%)	9 (9.09%)	0.001^3^
FFP transfusion amount (mL)	152.43 (322.96)	47.60 (167.12)	0.001^2^
Cryoprecipitate transfusion	4 (4.00%)	3 (3.00%)	0.700^3^
Cryoprecipitate transfusion amount (mL)	18.28 (110.27)	14.69 (125.73)	0.693^2^
Platelet transfusion	6 (6.00%)	2 (2.00%)	0.149^3^
Platelet transfusion amount (mL)	16.46 (79.20)	3.02 (30.00)	0.149^2^
Lactated ringers	99 (99.00%)	100 (100.00%)	0.999^1^
Lactated ringers amount (mL)	3297.83 (1759.02)	2402.88 (771.84)	< 0.001^2^
Saline	55 (55.00%)	23 (23.00%)	< 0.001^3^
Saline amount (mL)	456.00 (612.34)	131.50 (298.54)	< 0.001^2^
Hydroxyethyl starch 6%	38 (38.00%)	0 (0.00%)	< 0.001^1^
Hydroxyethyl starch 6% amount (mL)	533.50 (869.00)	0.00 (0.00)	< 0.001^2^
Albumin 5%	4 (4.00%)	67 (67.00%)	< 0.001^1^
Albumin 5% amount (mL)	27.72 (164.11)	832.75 (761.88)	< 0.001^2^
Total IV volume of colloids intraoperatively (mL)	1293.22 (1022.68)	1152.50 (689.19)	0.460^2^
Total IV volume of crystalloids intraoperatively (mL)	3758.83 (1850.67)	2546.08 (827.96)	< 0.001^2^
Day of surgery, fluids ≥1 L	40 (40.40%)	43 (43.00%)	0.710^3^

Continuous variables reported as mean (sd); categorical variables reported as frequency (percentage)

^1^Fisher exact

^2^Wilcoxon

^3^Chi-square

There was a decrease in estimated intraoperative blood loss from 1411 mL in the historical control group to 999 mL in the ERAS group, with a corresponding decrease in intraoperative packed red blood cell transfusion (945 mL vs 408 mL) and intraoperative fresh frozen plasma transfusion (152 mL vs 48 mL). There was a statistically significant decrease in intraoperative crystalloid use from 3756 mL in the historical group to 2546 mL in the ERAS group (*p* < 0.001) with no change in intraoperative colloid use (1293 mL vs 1153 mL) (Table 4).

The median length of stay decreased from 10 days (interquartile range (IQR) = 8–18) in the historical group to 7 ((IQR) = 6–11) days in the ERAS group (*p* < 0.0001). Readmission within 30 days was also lower in the ERAS group (38 vs 19%, *p* = 0.006) (Table 5).

**Table 5:** Outcomes

	Historical controls (*N* = 100)	ERAS patients (*N* = 100)	*p* value
Length of stay			
Median length of stay (days)	10 (8,18)	7 (6,11)	< 0.0001^1^
Mean length of stay (days)	14.86 (11.05)	10.00 (7.03)	< 0.001^1^
Recovery milestones			
Postoperative days to end IV	6.73 (7.67)	4.53 (4.40)	0.253^1^
Days to end IV ≥ 1	63 (63.00%)	77 (77.00%)	0.031^2^
Postoperative days to first stool	5.83 (3.83)	3.99 (1.93)	< 0.001^1^
Postoperative days to first liquid	5.05 (6.71)	1.09 (1.34)	< 0.001^1^
Postoperative days to first solid food	9.68 (8.79)	3.20 (2.52)	< 0.001^1^
Postoperative days to self-stoma management	10.11 (7.52)	8.88 (20.45)	< 0.001^1^
Postoperative TPN	32 (32.00%)	21 (21.00%)	0.078^2^
Postoperative days to end TPN	16.56 (7.72)	11.19 (6.93)	0.009^1^
Postoperative days to first on feet	1.98 (1.15)	1.25 (0.79)	< 0.001^1^
Opioids use and pain scores			
Intravenous opioids (total morphine equivalents)			
DOS	10.76 (30.72)	0.91 (6.05)	< 0.001^1^
POD1	36.09 (84.40)	8.66 (44.27)	< 0.001^1^
POD2	51.06 (122.05)	10.30 (55.44)	< 0.001^1^
POD3	50.17 (118.82)	7.51 (40.32)	< 0.001^1^
POD4	48.14 (116.83)	5.55 (34.69)	< 0.001^1^
POD5	38.21 (95.86)	1.94 (17.63)	< 0.001^1^
Total	234.45 (458.07)	34.87 (159.47)	< 0.001^1^
Maximum pain score			
DOS	3.49 (3.60)	3.27 (3.44)	0.711^1^
POD1	5.16 (3.23)	3.91 (3.13)	0.006^1^
POD2	4.40 (2.97)	3.60 (3.16)	0.049^1^
POD3	3.85 (2.98)	3.39 (2.84)	0.306^1^
POD4	4.01 (2.92)	3.79 (3.05)	0.642^1^
POD5	4.33 (3.08)	3.80 (3.23)	0.239^1^
Total	6.95 (2.67)	6.60 (2.76)	0.336^1^
Adverse events			
Readmission within 30 days	38 (38.00%)	19 (20.00%)	0.006^2^
Re-operation within 30 days	15 (15.31%)	9 (9.00%)	0.174^2^
Adverse event(s) at all during primary stay	87 (87.88%)	76 (76.00%)	0.030^2^
Complications during admission			
Respiratory	20 (20.00%)	17 (17.00%)	0.585^2^
Infectious	34 (34.00%)	20 (20.20%)	0.029^2^
Cardiovascular	25 (25.00%)	21 (21.00%)	0.502^2^
Postoperative ileus	65 (65.00%)	36 (36.73%)	< 0.001^2^
Surgical complication(s)	23 (23.23%)	15 (15.00%)	0.140^2^
Delirium	12 (12.37%)	2 (2.06%)	0.006^2^
PONV	72 (72.00%)	67 (67.68%)	0.506^2^
Requiring intensive care	10 (10.00%)	5 (5.00%)	0.179^2^
30 day mortality	2 (2.00%)	2 (2.00%)	0.999^2^

Length of stay reported as both median (IQR) and mean (SD). Median may be more representative due to outliers and non-parametric nature of outcome. Other continuous variables reported as mean (SD); categorical variables reported as frequency (percentage)

Respiratory complications include atelectasis, pneumonia, pleural fluid, respiratory failure, pneumothorax, and other unspecified

Infectious complications include wound infection, urinary tract infection, intraperitoneal or retroperitoneal abscess, sepsis, septic shock, and other unspecified

Cardiovascular complications include heart failure, acute myocardial infarction, deep venous thrombosis, pulmonary embolus, cardiac arrhythmia, cardiac arrest, and other unspecified

Surgical complications include anastomotic leak, urinary leakage, urinary tract injury, mechanical bowl obstruction, deep wound dehiscence, peritoneal soiling, and other unspecified

*TPN* total parenteral nutrition; postoperative days to end TPN measured among those who received postoperative TPN. *PONV* postoperative nausea or vomiting

^1^Wilcoxon

^2^Chi-Square

There were also significant reductions in time to important postoperative recovery milestones, including postoperative days to first stool (5.83 days vs 3.99 days, *p* < 0.001), to first PO liquid (5.05 days vs 1.09 days, *p* < 0.001), to first solid food (9.68 days vs 3.20 days, *p* < 0.001), to self-stoma management (10.11 days vs 8.88 days, *p* < 0.001), and days to first on feet (1.98 days vs 1.25 days, *p* < 0.001) (Table 5).

The ERAS group used dramatically less opioids overall and on each postoperative day with no change or a slight improvement pain scores. Total intravenous opioids through postop day 5 decreased from 234 ± 458 mg morphine equivalents in the historical group to 35 ± 159 mg morphine equivalents in the ERAS group (*p* < 0.001). Intravenous opioid use was less on the day of surgery and each postoperative day (all *p* < 0.001). There was a trend towards somewhat lower maximum daily pain scores on the day of surgery and each postoperative day although this only reached statistical significance on POD1 (5.16 vs 3.91, *p* = 0.006) and POD2 (4.40 vs 3.60, *p* = 0.049) and was not statistically different overall (6.95 vs 6.60, *p* = 0.336) (Table 5).

Overall complication rates were lower or unchanged in the ERAS group compared to the historical control group. The need for reoperation within 30 days was not statistically different (15% vs 9%, *p* = 0.174). The overall incidence of any adverse event during the primary stay decreased from 87 to 76% (*p* = 0.030). This high overall rate reflects the broad definition of any adverse event and the overall high morbidity of this operation and is not unexpected. There were statistically significant reductions in infectious complications (34% vs 20%, *p* = 0.029), postoperative ileus (65% vs 36%, *p* < 0.001), and delirium (12% vs 2%, *p* = 0.006). There were no statistically significant differences in respiratory complications (20% vs 17%, *p* = 0.585), cardiovascular complications (25% vs 21%, *p* = 0.502), surgical complications (23% vs 15%, *p* = 0.140), or PONV (72% vs 67%, *p* = 0.506). The number of patients requiring intensive care postoperatively was not statistically different (10% vs 5%, *p* = 0.179). Thirty-day mortality was unchanged (2% in each group, *p* = 0.999) (Table 5).

Average (mean) overall costs decreased 26.6% from $43,990.26 ± $28,496.09 per patient to $32,301.85 ± $18,756.60 per patient (*p* < 0.001). This overall reduction reflects a reduction in most individual domains with the biggest drivers being reductions in radiology, pharmacy, and intensive and intermediate nursing costs. There was a small increase in costs for outpatient clinics and no change in costs for a few domains (Table 6).

**Table 6:** Costs

	Historical controls (*N* = 100)	ERAS patients (*N* = 100)	*p* value
Labs	2378.84 (1183.74)	2123.44 (1094.39)	0.046^1^
Radiology	1742.60 (2636.01)	723.96 (971.44)	< 0.001^1^
Outpatient clinic	51.50 (106.67)	302.08 (251.84)	< 0.001^1^
PT/OT/speech services	833.29 (958.07)	498.54 (454.28)	< 0.001^1^
Pharmacy	5163.23 (5132.04)	3519.94 (2265.40)	0.044^1^
Surgery services	10,443.95 (4281.63)	10,954.26 (5739.40)	0.700^1^
Organ acquisition	2.62 (26.18)	94.34 (943.37)	1.000^1^
Blood products	1602.18 (1567.09)	930.27 (1056.96)	< 0.001^1^
Cardiology services	256.10 (729.06)	114.49 (279.73)	0.005^1^
Respiratory care	1410.74 (4826.77)	168.77 (387.66)	< 0.001^1^
Intensive care nursing services	6785.91 (9252.27)	3396.64 (5660.55)	< 0.001^1^
Intermediate care nursing services	12,379.30 (9111.22)	8836.85 (5924.26)	0.001^1^
Medical/surgical supplies	708.80 (1548.63)	346.38 (641.13)	< 0.001^1^
ER transport	18.41 (130.93)	8.22 (82.20)	0.566^1^
Other diagnostic and therapeutics	212.80 (704.33)	263.99 (1443.88)	0.062^1^
Other	0.00 (0.00)	19.69 (196.93)	0.322^1^
Total costs	43,990.26 (28,496.09)	32,301.85 (18,756.60)	< 0.001^1^

Continuous variables reported as mean (sd); all cost data reported in dollars

^1^Wilcoxon

In the supplementary analysis, overall time in target for SV (71.24% ± 28.17) was quite high, indicating good compliance with the goal-directed fluid therapy protocol. SV time in target did not have a significant correlation with LOS or other outcomes.

## Discussion

Radical cystectomy represents a significant surgical challenge to patients. The morbidity after radical cystectomy (with bilateral pelvic lymph node dissection and urinary diversion or bladder reconstruction) is between 30 and 64%, even in high volume centers (Cerantola et al. 2013; Shabsigh et al. 2009). ERAS pathways improve patient care, reduce morbidity, and shorten the length of stay (LOS); however, they are only starting to be utilized in major urologic surgery and have not been well studied in this setting (Cerantola et al. 2013).

Several small studies evaluating elements of the ERAS care pathways in radical cystectomy found benefits in postoperative morbidity, return to bowel function, or LOS (Roth et al. 2013; Jensen et al. 2015; Lee et al. 2014). A few studies of early fast track, multimodal, or enhanced recovery programs have also been published, showing improvements in length of stay, time to GI recovery, and post-operative ileus (Maffezzini et al. 2007; Arumainayagam et al. 2008; Pruthi et al. 2010; Bazargani et al. 2017; Daneshmand et al. 2014; Baack Kukreja et al. 2017). Adding anesthesia-related elements to an existing surgical enhanced recovery program reduced transfusions and nausea, while continuing to demonstrate good results in time to GI recovery and length of stay (Patel et al. 2018). Reviews and meta-analyses of these early studies have shown similar improvements in length of stay and return of bowel function with no change or an improvement in complications (Di Rollo et al. 2015; Mir et al. 2015; Tyson and Chang 2016).

In our study, median length of stay decreased from 10 days in the historical control group to 7 days in the ERAS group, to the benefit of patients who would rather be at home and to the benefit of the healthcare system in terms of cost and resource utilization. The mean length of stay was somewhat longer, as is common, driven by a few outliers with especially long lengths of stay, but also decreased from 15 to 10 days. Despite this shorter length of stay, readmission within 30 days also decreased in the ERAS group, indicating that these patients were truly recovering and ready for discharge.

Postoperative ileus is recognized as one of the major drivers of length of stay. Enhanced recovery programs target several factors which can contribute to ileus including preoperative fasting and bowel preparation, analgesic and anesthetic techniques, perioperative fluid management, nasogastric tube use, and postoperative diet restrictions. The most significant reduction in complications we saw was for postoperative ileus, the incidence of which was reduced from 65 to 34% and this may have been a major driver of the reduction in length of stay (Miller et al. 2014; Kehlet 2008).

Average overall costs decreased by 26.6% or $11,688.41 per patient. Some of these gains come from standardization of care and an overall reduction in time in the hospital with the corresponding reduction in resource utilization. As the two groups are separated in time, there are potentially other factors (such as changes in costs and hospital-wide practices over time) that may have contributed to this result. Reductions in costs are particularly important in light of the increasing emphasis on value-based care and the increasing use of bundled payments.

Overall crystalloid administered was reduced by 1212 mL or 32% in the ERAS group vs historical controls. Overall colloid use was unchanged although we did see a nearly complete shift in non-blood colloid selection from hydroxyethyl starch to albumin between the two time periods. Normal saline use also dropped dramatically in favor of balanced crystalloid solutions such as lactated ringers. Thirty-three percent of ERAS patients did not receive any colloid, implying that crystalloid was used for GDFT boluses or that other parameters indicated the patient was volume replete and did not require boluses (Table 4). Use of an advanced hemodynamic monitor increased dramatically from 13 to 99% (Table 4). While such monitors were available by provider request in the pre-ERAS period, there was no expectation of their use and they were not set up routinely in the ORs where cystectomies were performed. With introduction of the ERAS pathway, these monitors were brought to the OR and set up by default for cystectomy patients and providers were encouraged to make use of the monitor.

We did not find a significant correlation between SV time in target and LOS or most other outcomes. This is perhaps because time in target was high overall, indicating good compliance with the GDFT protocol, but not providing a lot of variability to offer additional predictive benefit. We did not have time in target data for the historical controls, and it is possible this would have been lower or more variable in this group.

While this is one of the more comprehensive studies of a complete ERAS protocol for radical cystectomy, there are some limitations. It was not a blinded randomized controlled trial (RCT). However, the comprehensive systems-based nature of implementing an ERAS program makes it nearly impossible to concurrently randomize some patients to receive it and some patients to receive standard (or historical) care and even harder to blind patients and providers to this randomization. ERAS programs implement many interventions simultaneously, limiting the ability to discern which interventions are most impactful. However, the individual interventions are evidence based when available and it is implementing multiple synergistic interventions that yield the best results.

While the data on the ERAS cohort was collected prospectively for this study, the data on the historical cohort was collected retrospectively by chart review and it is possible there was some recording bias or lack of specificity for certain outcomes (particularly the recovery milestones) for this retrospective group.

Another limitation is the long time frame of the study. This introduces the possibility of other shifts in practice patterns and hospital systems during the study period that may influence results. However, this is a necessary limitation to gather enough patients given the low frequency of radical cystectomy procedures, even at a major center. It also requires a significant amount of time to implement a system-wide ERAS program, necessitating some gap between the two groups.

## Conclusions

We found a significant reduction in length of stay associated with implementation of an enhanced recovery program for radical cystectomy. We also found a reduction in time to important recovery milestones, a reduction in some complications, and decreased costs associated with the procedure. Our data support the use of enhanced recovery protocols for radical cystectomy and add to the increasing body of literature supporting enhanced recovery for a wide variety of procedures. Future work will further refine enhanced recovery protocols, highlight which elements are most important, and expand the concept of enhanced recovery to a greater range of surgical procedures.

## Data Availability

The datasets used and/or analyzed during the current study are available from the corresponding author on reasonable request.
